# Identifying Major Transitions in the Evolution of Lithic Cutting Edge Production Rates

**DOI:** 10.1371/journal.pone.0167244

**Published:** 2016-12-09

**Authors:** Antoine Muller, Chris Clarkson

**Affiliations:** School of Social Science, The University of Queensland, St Lucia, Queensland, Australia; Université de Poitiers, FRANCE

## Abstract

The notion that the evolution of core reduction strategies involved increasing efficiency in cutting edge production is prevalent in narratives of hominin technological evolution. Yet a number of studies comparing two different knapping technologies have found no significant differences in edge production. Using digital analysis methods we present an investigation of raw material efficiency in eight core technologies broadly representative of the long-term evolution of lithic technology. These are bipolar, multiplatform, discoidal, biface, Levallois, prismatic blade, punch blade and pressure blade production. Raw material efficiency is assessed by the ratio of cutting edge length to original core mass. We also examine which flake attributes contribute to maximising raw material efficiency, as well as compare the difference between expert and intermediate knappers in terms of cutting edge produced per gram of core. We identify a gradual increase in raw material efficiency over the broad sweep of lithic technological evolution. The results indicate that the most significant transition in efficiency likely took place with the introduction of small foliate biface, Levallois and prismatic blade knapping, all introduced in the Middle Stone Age / Middle Palaeolithic among early *Homo sapiens* and Neanderthals. This suggests that no difference in raw material efficiency existed between these species. With prismatic blade technology securely dated to the Middle Palaeolithic, by including the more recent punch and pressure blade technology our results dispel the notion that the transition to the Upper Palaeolithic was accompanied by an increase in efficiency. However, further increases in cutting edge efficiency are evident, with pressure blades possessing the highest efficiency in this study, indicating that late/epi-Palaeolithic and Neolithic blade technologies further increased efficiency.

## Introduction

Technological efficiency is a key aspect of palaeoanthropological debates surrounding such topics as cognition, skill, intentionality, modernity, technological organisation and technological diversity [1–11]. It is commonly argued that innovations in lithic technology over the sweep of human evolution were accompanied by greater striking precision, longer reduction sequences, finer retouch, greater recursion and hierarchical planning, a greater variety of percussive and pressure flaking techniques, more intensive platform preparation, and predetermined and more standardised end-products [9, 10, 12–23]. These technological changes are also often viewed as existing in a feedback loop with biological evolution that drove dexterity, cognition, and syntactic language [9, 23–33]. Within this narrative, blade and microblade technologies are often depicted as the pinnacle of evolution in core technology and a key component of the ‘Upper Palaeolithic Revolution’, involving highly standardised blank production and careful preparation and maintenance of core volume and efficiency [3, 34–41].

Of particular concern to this study is this pervasive assumption that blades offer greater efficiency in cutting edge production [35, 36, 42–48], underpinned by an early experiment examining the efficiency in edge production of pressure blade cores [40]. Those who are not convinced of the gains in efficiency offered by blade production cite the raw-material wastage involved in selecting high-quality stone required for successful blade manufacture, the higher risk of critical breakages owing to the thinness of blades, and the fewer opportunities for retouch events due to the narrowness of blades [49–51].

Stone knapping technologies are often portrayed as evolving in a linear fashion, described by Clark [52] as a series of ‘modes’. The sequence begins with the single and multiplatform cobble industries of the Oldowan (Mode 1) at c.2.6 million years ago (mya), developing into bifacial and discoidal technologies (Mode 2) of the Early Stone Age/Lower Palaeolithic after c.1.6 mya. These were followed by the first appearance of Levallois (Mode 3) in the Middle Stone Age/Middle Palaeolithic, the development of blade technology (Mode 4) in the Upper Palaeolithic, and finally the appearance of the microlithic industries (Mode 5) of the Later Stone Age and Mesolithic. Despite the popularity of this scheme, it is now clear that technological evolution is far from linear, but is instead multidirectional, branching and recursive. For example, blade technology is securely dated to well before the Upper Palaeolithic [53–57], is not confined to anatomically modern humans, and appears and disappears in many regions over time [49, 58–60].

Several experiments over the last four decades have compared raw material efficiency for a range of core reduction strategies [17, 40, 50, 61–64], most of which consider the efficiency of blade core reduction. All bar one [40] of these experiments have called into question the supposed advantages in efficiency afforded by blade technology. Despite these findings, these experiments typically involve a comparison of only two reduction strategies such as biface versus blade or discoidal versus blade for example. For this reason, this paper compares the efficiency of eight core reduction strategies (bipolar, multiplatform, discoidal, biface, Levallois, prismatic blade, punch blade and pressure blade), which are common throughout the span of human evolution. We therefore provide the most comprehensive study of raw material efficiency to date. Previous experiments have also employed varied methodologies, hampering direct comparison of results. We therefore adopt the method of computer analysis developed by Eren et al. [61] and refined by Heighway [17] to measure cutting edge to mass ratios.

### Cutting Edge Efficiency

A key component of technological efficiency is lithic raw material efficiency, or the extent to which different knapping technologies and techniques conserve core mass during production. In this present paper, raw material efficiency is evaluated by calculating cutting edge efficiency, or the length of possible cutting edge per gram of original core. Sheets and Muto [40] first explored cutting edge efficiency by demonstrating the efficiency of pressure blades. Their method for calculating the cutting edge length, by measuring the length of the blade and doubling the result, was inaccurate considering that length measurements do not account for wavy or tapering blade edges, and their assumption of blade symmetry introduces a high degree of error.

More recently, some have sought to experimentally assess the raw material efficiency of biface reduction [62–64]. Rasic and Andrefksy [64] and Jennings et al. [62] compared blade cores to bifacial reduction, finding parity in their raw material efficiency. These analyses did not include a consideration of cutting edge length however, focussing instead on blank count, size and shape. Cutting edge length was considered in a study by Prasciunas [63], who found bifacial and multiplatform reduction to be equivalently efficient when considering blanks only larger than 5g. While each of these experiments highlight the efficiency of bifaces, and bring into question the supposed advantages in efficiency afforded by blade reduction, the variety of methods and units of measurement used to assess raw material efficiency hamper comparisons among these experiments, and between the earlier work of Sheets and Muto [40]. Additionally, the use of a range of percussor types, such as soft and hard hammers, or hammers of different sizes, limits the reproducibility of these studies as different percussors can influence core and flake morphology [65].

Brantingham and Kuhn [1] applied geometric models to Levallois core reduction and found that the nature of Levallois reduction is geared towards minimising waste and maximising productive output. We therefore include Levallois flaking in our experimental sample to test this hypothesis in relation to the other technologies and situate it in the broad sweep of technological evolution.

Another methodologically rigorous approach to raw material efficiency was conducted by Eren et al. [61], who compared the cutting edge length per original core mass of prismatic blade technology against discoidal technology, finding no significant difference between blade and discoidal cores. The hypothesis of Chazan [50] that wider flakes can more frequently be resharpened thereby extending their use-life was also tested by Eren et al. [61], who found that when the potential for further retouch events is considered, discoidal reduction is more efficient in terms of cutting edge per gram of core than blade reduction. Also of interest to this present study is the highly precise and reproducible method of Eren et al. [61], who measured cutting edge length by reducing photographs to complex polygons and employing software to calculate the edge length. In the interests of reproducibility and comparability of results, this approach is also adopted here.

While Eren et al. [61] set out to examine the transition to the Upper Palaeolithic using prismatic blade technology, more recent dates situate the advent of this technology well before the Upper Palaeolithic [53–57]. Our sample includes punch and pressure blade technology, which hitherto have only been dated to the Upper Palaeolithic and onwards. Thus, while Eren et al. [61] were in effect comparing the Lower to Middle Palaeolithic transition, we offer the first real examination of cutting edge efficiency beyond the Middle Palaeolithic and into the Upper Palaeolithic, Epipalaeolithic and Neolithic (also including late Mesoamerican technologies). Moreover, while all previous attempts at examining raw material efficiency compared no more than two technologies, we compare eight technologies that broadly represent the evolution of core technology from the Oldowan to the Neolithic. As these previous studies found raw material efficiency to be equivalent among bifaces and prismatic blade cores [62, 64], bifaces and multiplatform cores [63], and prismatic blades and discoidal cores [61], we seek to test the null hypothesis that no significant differences in cutting edge per gram occur among the eight different reduction strategies under investigation.

### Causes of Variability in Efficiency

A previous attempt at identifying the features of a flake which maximise its usable edge per unit of volume was conducted by Lin et al. [66], who found that increasing the ratio of length to width (elongation), decreasing flake thickness relative to surface area, and decreasing platform size, could all contribute to maximising the efficiency of individual flakes. Moreover, they argue that these features can be maximised for flakes by decreasing platform depth and increasing exterior platform angle (EPA). The large sample produced in this present study allows for a consideration of the role of these, and other, flake attributes in altering cutting edge efficiency and the tendency of different common and well-known reduction sequences to increase cutting edge efficiency by emphasising these features.

### Skill

We additionally examine the effect of knapping skill on the efficiency of reduction sequences, a divergence from previous knapping skill studies which typically focus on core reduction ability. Previous approaches to knapping skill include considerations of the presence of successes or failures in the knapping sequence [4, 67, 68], experimental attempts to identify markers of knapping skill in the individual [69], ethnographic reconstructions of complex knapping sequences [70], and analyses of the effect of raw material quality on knapping skill [71, 72]. In experiments and the archaeological record, successive step or hinge terminations, overshot flakes, flakes with an undesired morphology, percussor marks attempted too far from the platform edge or on platforms of unsuitable angles have all been used as evidence of comparatively unskilful knapping [4, 8, 68, 69, 72–76]. Of particular interest to this study is that cores knapped by novice or intermediate knappers tend to have a higher rate of unsuccessful flake removals and produce flakes of smaller size [8, 11, 73, 74, 76]. The influence of this discrepancy between intermediate knappers and experts on cutting edge efficiency will be explored in this study.

## Materials and Methods

### Knapping Experiments

A total of 44 cores were knapped in this experiment to determine the efficiency of each reduction strategy. Raw material efficiency was estimated here based on the length of resulting cutting edge relative to original core mass. While the number of blanks, mass of blanks and mass of waste were all recorded, it is the cutting edge per gram of original core values that offer the most meaningful comparisons among the different technologies. The length of cutting edge offers a quantification of the amount of usability possessed by a flake, and by reducing this edge length to a ratio of core mass any variation in beginning core size is negated. Of course, the function of this edge length is not limited to cutting alone, as sharp edges afforded by lithic technology have been used, among other things, for chopping, sawing, scraping and piercing. As Eren et al. [61] already examined the role of use-life on cutting edge efficiency, we consider efficiency in terms of cutting edge length per gram of original core for unretouched flakes only.

The sample of technologies examined in this study includes bipolar, multiplatform, discoidal, bifacial, Levallois, prismatic blade, punch blade and pressure blade technology. These eight technologies span much of the evolution of lithic technology and encompass many species of hominin knappers. While not all hominins may conceive efficiency and optimality equally, if at all, it is not hominin intentionality we wish to examine. Rather, this method is aimed at identifying temporal transitions in cutting edge efficiency regardless of whether improvements in efficiency were deliberate.

As we also seek to evaluate the role of knapping skill on cutting edge efficiency, both expert and intermediate knappers were involved in most of these reduction sequences. The expert knapper had approximately two decades of experience in stone knapping, while the intermediate knapper had only a few years of experience but could adequately reproduce technologies like Levallois and prismatic blade reduction. The expert knapper reduced two cores and the intermediate knapper reduced five cores for the multiplatform, discoidal, biface, Levallois and prismatic blade technologies. The intermediate knapper did not conduct the bipolar portion of the experiment as this technology requires such little skill that minimal variation in cutting edge efficiency was expected. Similarly, punch and pressure blade knapping requires such a high level of skill that it could be executed only by the expert knapper. For each of these three technologies, the expert knapper conducted three repetitions.

The results produced from both the intermediate and expert knapper are included in all analyses of cutting edge efficiency in an attempt to capture the broad spectrum of skill among past hominin knappers. Including only an expert *Homo sapiens* knapper would not adequately summarise the millennia of evolution in brain [23, 24, 30, 77] and hand [78–80] morphology that influences the cognition and skill of different knappers in the past and present.

All nodules used in this experiment were of the same highly cryptocrystalline Texan flint, possessing very rare and minor impurities. Any nodules with heat damage or critical impurities were immediately discarded and replaced. The starting nodules weighed approximately 700g (Table 1), with the exception of the three bipolar cores that were far smaller owing to the fact that bipolar reduction typically occurs only on small cores. A Kruskal-Wallis test reveals that there is no significant difference between the mean of core masses for any reduction strategy other than bipolar (*H* = 3.18; *df* = 6, 34; *p* = 0.78). All flakes for the multiplatform, discoidal, biface, Levallois and prismatic blade reductions were detached using the same standardised copper-headed billet weighing approximately 140g. This modern billet was used in favour of more traditional billets as the mass and hardness of copper is analogous to soft stone, antler or wood [40, 65, 81, 82], and the copper billet provided a constant and standardised shape throughout all experiments. Refer to S1 Text (as well as S1 Dataset) for a test of the suitability and efficacy of using copper billets as an analogue for the wide range of soft hammers available to prehistoric knappers. Bipolar knapping was conducted with a hard stone anvil, and punch and pressure knapping was conducted with different copper headed billets owing to the specific requirements of these reduction sequences. All knapping debris was collected for later analysis.

**Table 1:** Mass values of the bipolar, multiplatform, discoidal, biface, Levallois, prismatic blade, punch blade and pressure blade cores, waste and blanks from each reduction sequence. Initial nodule masses in bold refer to reduction sequences conducted by the expert knapper.

Core	Initial Nodule (g)	Exhausted Core (g)	Waste Chips (g)	Total Blanks
Bipolar	**119**	4.54	12.41	22
	**134**	4.08	16.13	25
	**152**	35.90	29.01	28
Multiplatform	**722**	13.5	50.8	77
	**766**	17.0	43.7	57
	720	67.9	145.7	100
	704	37.1	139.3	96
	690	12.4	68.1	150
	740	23.2	318.5	101
	725	24.9	148.1	143
Discoidal	**790**	17.5	89.5	99
	**772**	9.0	75.2	121
	741	46.3	104.6	100
	709	39.7	167.1	98
	690	44.7	140.2	90
	730	137.8	235.8	90
	712	55.0	165.4	145
Biface	**747**	32.42	187.53	226
	**786**	34.77	150.7	220
	757	13.19	152.96	172
	703	20.92	124.32	198
	716	7.83	99.73	156
	754	29.15	120.88	172
	677	28.01	103.96	194
Levallois	**758**	10.8	142.7	134
	**724**	15.1	122.5	147
	712	76.7	176.9	167
	740	48.7	171.8	123
	704	172.1	270.8	98
	735	41.2	123.9	130
	712	40.5	184.7	139
Prismatic Blade	**716**	11.6	135.7	144
	**735**	15.8	125.2	168
	740	192.3	279.9	125
	718	78.2	183.2	131
	734	78.8	143.1	120
	747	72.1	145.8	121
	695	76.7	167.3	132
Punch Blade	**726**	85.35	54.13	156
	**778**	63.35	82.84	214
	**748**	101.62	51.54	175
Pressure Blade	**753**	48.62	85.74	211
	**755**	74.38	85.33	226
	**712**	30.10	49.94	189

Throughout the experiments reduction continued until the cores became exhausted and no more blanks could be removed, whether due to small core size, high platform angles, accumulated step or hinge terminations, or a combination of these factors. Although Eren et al. [61] measured only formal blades and discoidal flakes while disregarding the products of core reshaping, here we define blanks as any removed flake larger than 2cm. We adopted this size threshold, as flakes larger than 2cm can easily be manipulated in the hand for tool use [83]. Additionally, we sought to avoid complications arising from assuming knowledge about past knappers’ intentionality, particularly surrounding which removals they desired over which removals they considered waste. This point is particularly important for the biface iterations of this experiment, as the core itself is typically the desired end-product, whereas with all other knapping technologies examined here the core is generally considered waste. However, there is no *a priori* reason that prehistoric knappers would not have used the flakes produced from any form of core shaping. We are therefore measuring the maximum potential efficiency of each reduction sequence. This arbitrary threshold also allows for greater reproducibility of results, compared with methods that rely on subjective decisions regarding what constitutes a blank.

### Reduction Sequences

In order to maintain experimental control, both the expert and intermediate knappers adhered to strict reduction sequences, reconstructed from archaeological, ethnographic and experimental sources. This section outlines the archaeological correlates of each of the eight reduction technologies and highlights the methodological aspects key to successfully accomplishing these technologies.

Originating in the Oldowan, but with perhaps even older roots at Lomekwi 3 in Kenya [75], bipolar knapping is one of the oldest stone tool technologies and is executed by positioning a core on an anvil and striking the exposed platform until a flake is detached. Bipolar knapping proceeded in this relatively expedient fashion by exposing and striking new platforms until the cores were exhausted, following archaeological and ethnographic examples [4, 84–90].

Multiplatform reduction was conducted in this experiment via expedient and opportunistic selection of suitable platforms involving no constraints on the direction from which a flake can be removed. This sequence was reconstructed from a range of archaeological correlates [91–94]. With its origins in the Oldowan, the primary aim of multiplatform knapping is the production of as many large and usable flakes as possible, while not creating too high or too low edge angles that would inhibit further reduction.

Discoidal knapping involved the formation of a core with a bi-conical morphology, created via bifacial and radial flake removals. In order to maximise the use-life of the discoidal cores, both knappers intended each flake to both maintain this specific morphology as well as expose new suitable platforms. To maximise the utility and applicability of these results, the discoidal reduction sequences were modelled on well described reduction sequences [61, 95–97], as well as archaeological examples from a range of regions and time periods [4, 5, 93, 98–102].

With its roots in the Acheulean, bifacial knapping is an enduring and widespread technological innovation. However, in the interests of maintaining similar original nodule size and allowing the knapper to exploit the core until near exhaustion, as was the case with all other technologies, more recent and more heavily reduced bifacial technology is examined in this study. Reduction proceeded following archaeological examples of small foliate bifaces from the African Middle Stone Age [103–106] and the European Middle Palaeolithic [107–111]. Thin and invasive flakes were removed from both faces of the core, maintaining a sharp plane of intersection between the equivalent hemispheres.

Recurrent Levallois knapping, ubiquitous in the Middle Stone Age or Middle Palaeolithic, was conducted via establishing with radial flaking two asymmetrical hemispheres, one relatively flat upper hemisphere and one more protruding lower hemisphere. Meanwhile, the final platform was carefully faceted on the lower hemisphere. Following known reduction sequences [1, 19, 96, 97, 112–115], and archaeological examples [71, 116–123], convexities were rigorously maintained on the upper surface in order to control the morphology of the recurrent Levallois flakes. These convexities were steepened or flattened with short dihedral flakes or invasive flakes respectively, with the intention of allowing the applied force to the faceted platform to remove a large portion of the upper surface without overshooting the core. This process of establishing two hemispheres and a faceted platform was repeated until no more recurrent Levallois flakes could be removed.

Prismatic blade core production in this experiment involved establishing a strong and flat, or slightly concave, platform from which to remove as many long and thin blades as possible. Following several archaeological examples [55–57, 97, 124–126], blades were removed by striking the platform above a long and strong ridge on the core surface. Each successive blade removal created two new ridges at the intersection of flake scars, from which subsequent blades could be removed. Owing to the desire for long and thin flakes in blade reduction, overhang removal and abrasion is a particularly important aspect of this type of core reduction and was frequently conducted by the knappers. While this experiment involves unidirectional prismatic blade core knapping, bidirectional removals were at times used to maintain the core surface morphology or correct and straighten any haphazard ridges.

A variation of blade technology that occurred in the Upper Palaeolithic and onwards is the punch blade technique, in which one end of an intermediary tool, or ‘punch’, is placed on the core’s platform while the other end is struck by the percussor. This form of indirect percussion allows the knapper to situate the punch very close to the platform edge immediately above a ridge, thereby ensuring the precise placement of each blow. Reduction proceeded in this experiment using a copper-tipped punch and by following experimental and archaeological examples from Mesoamerica, Europe and the Near East [65, 81, 127–133].

Another blade technology of the Upper Palaeolithic and onwards is pressure blade manufacture, involving applying pressure from an indentor rather than using direct or indirect percussion. Like punch blade technology, the indentor can be very accurately placed, allowing greater control of blade production. The pressure blade component of this experiment involved a chest crutch and was conducted following extensive experimental and archaeological correlates [40, 132, 134–137].

### Flake Measurements

**Fig 1:**
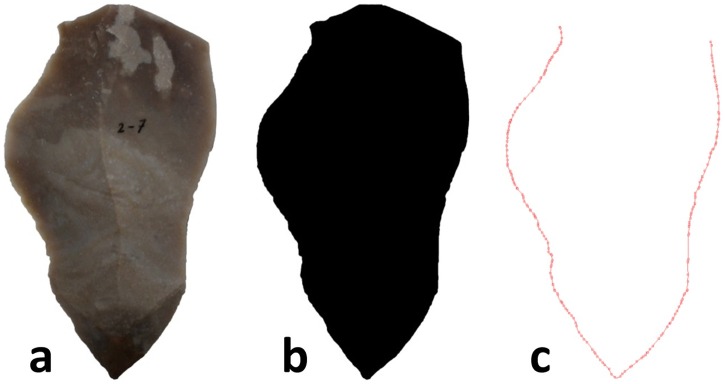
Demonstration of the method used to measure cutting edge length, showing a photograph of an original blank (a), and two stages in the process of reducing the photograph to a measurable polygon (b and c). Note the platform is excluded in the polygon measurement so as to measure possible cutting edge only.

Due to the varied morphology of flakes, calliper measurements of cutting edge can be highly inaccurate. Therefore, cutting edge length was determined by measuring the outline of digital photographs of flakes placed ventral side down on a flat surface. Following the methodology of Eren et al. [61] and Heighway [17], each blank larger than 2cm was photographed alongside a scale-bar using a digital camera. These images were imported to Adobe Photoshop CC and scaled to actual blank size, then reduced to a polygon in Adobe Illustrator CC (Fig 1). This software was used to automatically trace the polygon’s perimeter and calculate the edge length in millimetres. Platforms and broken or dull edges were excluded from the perimeter measurement as they do not serve as a suitable cutting edge.

Finally, in order to explore the possible reasons for any variation in the efficiency of the eight reduction sequences under examination, all complete and formal blanks were collected from each of the expert’s reduction sequences. These flakes were then weighed and measured using digital scales, callipers and a goniometer. The mass, dimensions (including length, mean width, mean thickness, platform width and bulb thickness), exterior platform angle (EPA), platform type, termination type, initiation type and platform preparation type were recorded for each flake. Mean flake width was calculated by averaging three equally spaced width measurements, proximal width, medial width and distal width, in order to encompass any irregular edge morphology. Similarly, flake thickness was assessed by averaging five thickness measurements taken at regular intervals on the flake. Bulb thickness was measured by subtracting the thickness of the flake at the apex of the bulb of percussion by the thickness of the flake immediately below the bulb of percussion, while accounting for any amorphous dorsal morphology. These measurements were taken to allow an exploration of the effects of flake size and shape on the cutting edge efficiency.

## Cutting Edge Efficiency

Throughout the 44 reduction sequences, a total of 30.40kg of flint was knapped, producing 5930 blanks with a cumulative cutting edge length of 613.53m. Table 2 shows the total values for each reduction strategy, summarising mass, count and cutting edge results, with a Kruskal-Wallis test for equal medians exploring the variability among the different reduction sequences.

**Table 2:** Mean mass, counts and cutting edge values for each reduction strategy. Kruskal-Wallis tests were conducted for each variable based on the values of the three or seven repetitions of each reduction method. Variables containing significant differences among the eight different technologies at the α = 0.05 level are represented in bold. *The bipolar values were not included in the first five statistical comparisons, as significantly smaller cores were used owing to the typically small size of bipolar cores.

Reduction Method	Mean initial nodule mass (g)*	Mean number of blanks*	Mean mass of all blanks (g)*	Mean mass of waste (g)*	Mean cutting edge (mm)*	Mean cutting edge per gram of core (mm/g)
Bipolar (N = 3)	135.14	25.00	101.11	34.02	1404.33	10.36
Multiplatform (N = 7)	723.86	103.43	565.28	158.60	12167.06	16.90
Discoidal (N = 7)	734.86	106.14	545.17	189.69	12995.91	17.69
Biface (N = 7)	734.29	191.14	576.45	158.05	14244.75	19.39
Levallois (N = 7)	726.43	134.00	498.09	228.34	15857.12	21.83
Prismatic Blade (N = 7)	726.43	134.43	482.76	243.67	16303.79	22.46
Punch Blade (N = 3)	750.67	181.67	604.38	146.28	17236.65	22.95
Pressure Blade (N = 3)	740.00	208.67	615.24	124.70	18875.14	25.91
Kruskal-Wallis	H = 3.18	**H = 28.69**	H = 10.53	H = 9.55	**H = 16.33**	**H = 22.92**
	df = 6, 34	**df = 6, 34**	df = 6, 34	df = 6, 34	**df = 6, 34**	**df = 7, 36**
	p = 0.78	**p < 0.001**	p = 0.10	p = 0.15	**p = 0.012**	**p = 0.0018**

**Fig 2:**
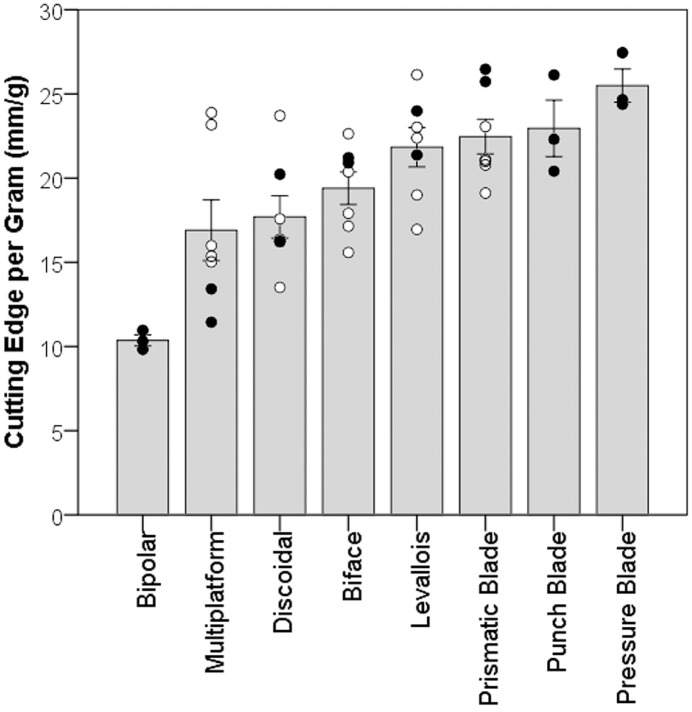
Bar chart, with one standard error bars and each data point superimposed, showing the cutting edge per gram values for each repetition of bipolar (*N* = 3; *μ* = 10.36), multiplatform (*N* = 7; *μ* = 16.90), discoidal (*N* = 7; *μ* = 17.69), biface (*N* = 7; *μ* = 19.39), Levallois (*N* = 7; *μ* = 21.83), prismatic blade (*N* = 7; *μ* = 22.46), punch blade (*N* = 3; *μ* = 22.95) and pressure blade (*N* = 3; *μ* = 25.49) knapping. Open circles represent the results from the intermediate knapper and closed circles represent the expert knapper.

While significant differences occur among the blank counts (*H* = 28.69; *df* = 6, 34; *p* < 0.001), Mann-Whitney tests with Bonferroni corrections (counteracting the increased risk of a type-I error during multiple comparisons) reveal that, excluding the bipolar reductions, the only significant difference is that the biface reduction sequences produced significantly more blanks on average than the multiplatform and discoidal repetitions (*U* = 24.5; *p* = 0.045 for both). This discrepancy is likely explained by the typically higher fragmentation rates that accompany biface reduction. Bifacial knapping involves the concerted production of very thin, expanding flakes called thinning flakes, which increases the likelihood of breakage. This means however, that the number of flakes produced is unlikely to be an adequate representation of raw material efficiency. Instead we turn to the length of cutting edge produced per gram of core to assess raw material efficiency among the sample of eight knapping technologies. Table 2 shows the total cutting edge per gram for each reduction strategy, with an ascending trend through this order of reduction sequences. Fig 2 explores this pattern further, by plotting the total cutting edge per gram for each repetition. This ascending trend is further accentuated when considering the expert reduction sequences only (closed circles). The implications of this pattern will be discussed in the ‘Skill’ section below.

**Fig 3:**
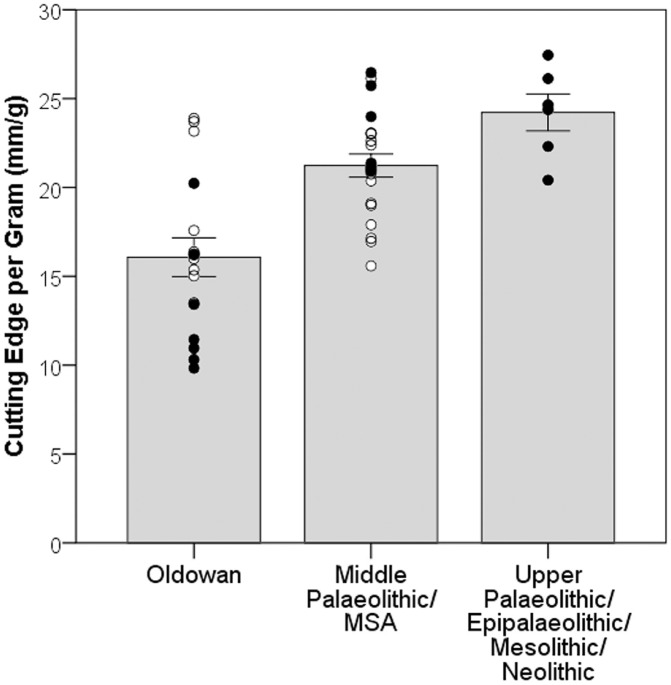
Bar chart, with one standard error bars and each data point superimposed, of the eight technologies grouped into their corresponding time periods, showing the Oldowan, consisting of bipolar, multiplatform and discoidal technologies (*N* = 17; *μ* = 16.07), the Middle Palaeolithic, consisting of biface, Levallois and prismatic blade technologies (*N* = 21; *μ* = 21.23), and the Upper Palaeolithic and onwards, consisting of punch blade and pressure blade (*N* = 6; *μ* = 24.22). Open circles represent the results from the intermediate knapper and closed circles represent the expert knapper.

A Kruskal-Wallis test reveals that significant differences occur among the different reduction strategies in terms of cutting edge per gram (*H* = 22.92; *df* = 7, 36; *p* = 0.0018), however the subsequent Mann-Whitney pairwise post-hoc analysis with Bonferroni corrected *p*-values returned no significant results. This means that no individual knapping strategy was significantly more efficient than another. With the original Kruskal-Wallis test suggesting that significant differences do occur among the samples, the variability among the eight different technologies was examined further by combining each technology into broad time periods reflecting their first documented production in the archaeological record. These were the Oldowan (bipolar, multiplatform and discoidal), Middle Palaeolithic (biface, Levallois and prismatic blade) and Upper Palaeolithic and onwards (punch blade and pressure blade) (Fig 3). Lithic technologies are not produced in isolation of course. Prehistoric toolkits would have consisted of varying proportions of the available technologies at the time, depending on raw material availability and prospective function. Therefore, combining these eight technologies into broad time periods will allow for more meaningful comparisons of broad-scale temporal trends in cutting edge efficiency. Ascribing these technologies to different time periods is done cautiously however, as our knowledge of evolution in lithic technology is being constantly revised. It is also acknowledged that lithic technologies often disappear and reappear at certain times, as well as undergo modification. That is why we ascribed particular technologies to these time periods according to their earliest known use. If certain technologies receive older or younger dates, then the following analysis could easily be updated to reflect any changes.

Significant differences occur among these three grouped samples (*H* = 17.13; *df* = 2, 41; *p* < 0.001), with a Mann-Whitney pairwise test with Bonferroni corrected p-values revealing that the Middle Palaeolithic and Upper Palaeolithic reduction sequences produced a significantly greater length of cutting edge per gram of original core compared with the Oldowan technologies (*U* = 64; *p* = 0.0025 and *U* = 6; *p* = 0.0055 respectively). Despite a difference of more than 3mm/g of cutting edge length per gram of core between the Middle and Upper Palaeolithic and onwards technologies, this difference is not significant (*U* = 30; *p* = 0.17). These results reveal that the transition from Lower to Middle Palaeolithic toolkits was accompanied by an increase in the efficiency of cutting edge production per mass of core. On the other hand there appears to be no inherent increase in cutting edge efficiency at the transition from the Middle to Upper Palaeolithic. Again, this pattern is accentuated when considering expert knappers only (closed circles), with little difference observable at the Middle to Upper Palaeolithic transition.

Interestingly, pressure blades outperformed all other core technologies tested in this experiment. While the technologies examined here that formed a component of the Upper Palaeolithic and onwards are by no means significantly more efficient than the preceding period, it would appear that the evolution of cutting edge efficiency that is evident by the ascending trend in Fig 2 continued during the Upper Palaeolithic, Epipalaeolithic, Mesolithic and Neolithic.

## Causes of Variability in Efficiency

**Fig 4:**
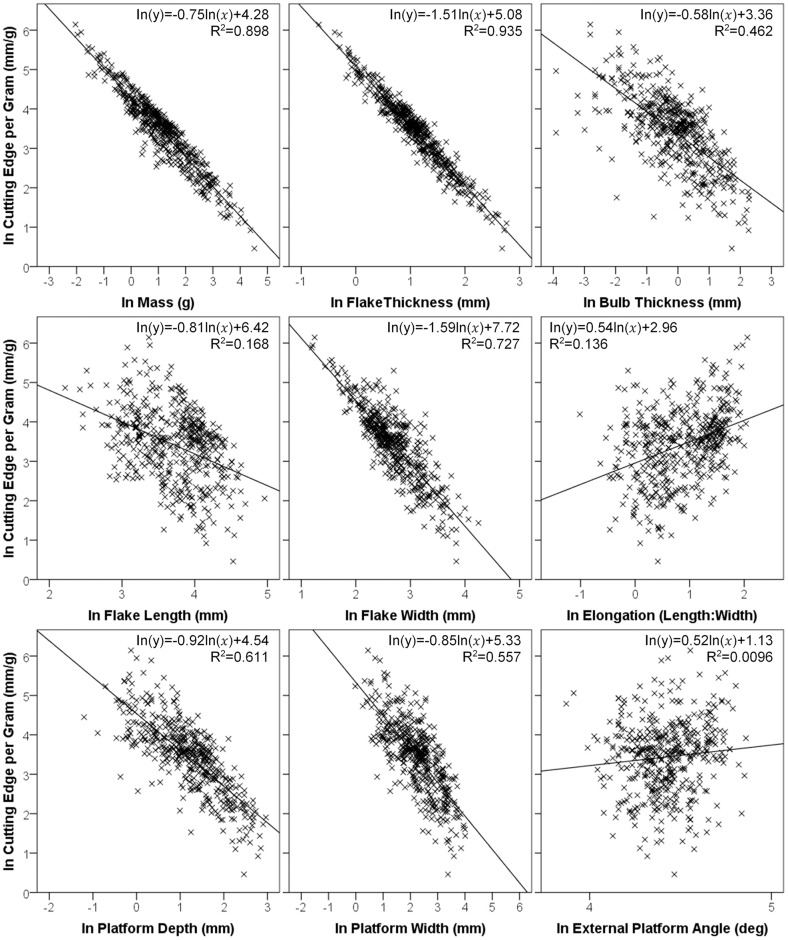
Scatter plots with both axes transformed using the natural log (ln) examining the influence of mass, thickness, bulb thickness, length, width, elongation, platform depth, platform width and exterior platform angle (EPA) on the cutting edge length per gram of core for individual flakes. The sample size of each scatter plot is 488, except for the platform depth, platform width and EPA scatter plots, which had sample sizes of 460 owing to the presence of some crushed platforms.

Having examined the broad temporal trend in cutting edge efficiency, we now turn to the individual flake attributes which contribute to this variability. Based on measurements from the sample (*N* = 488) of complete and formal flakes produced by the 19 experimental reduction sequences conducted by the expert knapper, we can identify features of flakes which maximise flake economy. Fig 4 plots cutting edge length per gram against nine flake attributes, most of which reveal power relationships between the axes. To present these trends more clearly, both axes for all nine charts were transformed to linear relationships using the natural log (ln). From these charts, it is clear that minimising flake mass (*R*^*2*^ = 0.898), flake thickness (*R*^*2*^ = 0.935), bulb thickness (*R*^*2*^ = 0.462), flake width (*R*^*2*^ = 0.727), platform depth (*R*^*2*^ = 0.611) and platform width (*R*^*2*^ = 0.557) all strongly contribute to maximising the cutting edge length per gram of individual flakes. These results partly confirm the findings of Lin et al. [66], who found reducing flake thickness, bulb thickness and platform size had a positive effect on flake economy.

Where our findings diverge is in the role of elongation (length divided by width) and EPA. Lin et al. [66] used geometric models and flake measurements to hypothesise that increasing the ratio of length to width and EPA should maximise the economy of flakes. Our findings suggest, however, that flake length (*R*^*2*^ = 0.168) and elongation (*R*^*2*^ = 0.136) had very little influence on the efficiency of the flakes. Increasing length relative to width had only a very weak impact on cutting edge length per gram, which was far superseded by other size attributes like minimising thickness and width. It is interesting therefore that the three blade technologies, all of which maximise elongation, were the most efficient at cutting edge production. This could largely be credited to the production of narrow and thin blades within these knapping schemas, rather than the elongate nature of blades. The weakly positive relationship between elongation and cutting edge per gram is likely explained by what Lin et al. [66] identify as the ‘square cube principle of proportional solids’, whereby increases in the surface area of an elongate flake results in a lesser increase in volume compared with a more circular flake. Lastly, EPA, which was identified as a fundamental component of flake economy by Lin et al. [66], appears in this present study to have a negligible (*R*^*2*^ = 0.0096) impact on cutting edge efficiency. Lin et al. [66] used EPA as a proxy measure for efficiency as it influences flake morphology. Interestingly, our results suggest that optimising EPA is only one of several ways of increasing cutting edge efficiency.

**Fig 5:**
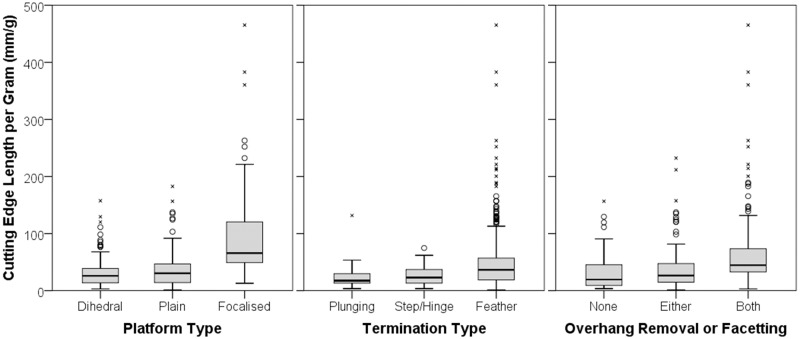
Boxplots of qualitative variables. Platform type plot (a) compares dihedral (*N* = 178; *Mdn* = 25.99), plain (*N* = 182; *Mdn* = 30.52) and focalised (*N* = 100; *Mdn* = 65.81) platforms. Termination type plot (b) compares plunging (*N* = 22; *Mdn* = 17.45), step or hinge (*N* = 58; *Mdn* = 23.02) and feather (*N* = 408; *Mdn* = 36.45) terminations. Platform preparation plot (c) compares no preparation (*N* = 105; *Mdn* = 19.49), either overhang or faceting (*N* = 202; *Mdn* = 26.44) and both overhang and faceting (*N* = 181; *Mdn* = 44.67).

In terms of the qualitative features of flakes, Fig 5 shows boxplots of the cutting edge length per gram of each flake according to different platform, termination and platform preparation types. A Kruskal-Wallis test for equal medians reveals that platform type has a significant impact on the production of cutting edge length per gram of core (*H* = 125.5; *p* < 0.001). Mann-Whitney tests with Bonferroni corrections reveal that flakes with focalised platforms have significantly greater cutting edge length per gram than those with dihedral (*U* = 2080; *p* < 0.001) or plain (*U* = 14820; *p* < 0.001) platforms. Termination type also significantly influences cutting edge efficiency (*H* = 22.9; *p* < 0.001), with feather terminations facilitating higher cutting edge per gram of core than plunging (*U* = 2517; *p* = 0.002), or step and hinge (*U* = 8426; *p* = 0.001) terminations. Finally, platform preparation strategies are similarly effective at increasing cutting edge efficiency (*H* = 74.22; *p* < 0.001), with the use of either overhang removal or faceting resulting in significantly higher cutting edge per gram than flakes without platform preparation (*U* = 8803; *p* = 0.044). Additionally, flakes exhibiting both overhang removal and faceting performed significantly better than those without preparation (*U* = 4627; *p* < 0.001), as well as those with only one type of preparation (*U* = 10550; *p* < 0.001). Attributes like platform, termination and platform preparation type are all associated with the quantitative associations above. For example, focalised platforms, feather terminations and extensively prepared platforms all contribute to producing flakes with low thickness, bulb thickness and flake width values.

These analyses offer a holistic identification of the features that make a flake efficient in terms of the cutting edge length produced per gram of original core. In summary, it appears that the efficiency of flakes are negatively impacted by areas of mass on a flake that do not contribute to the cutting edge, such as a bulb or amorphous dorsal surface, as well as portions of the flake perimeter that do not contribute to the cutting edge, such as platforms or steep broken edges. The most efficient flakes, therefore, are those that are thin and narrow, with diffuse bulbs, small platforms, feather terminations and extensive platform preparation.

Throughout the evolution of lithic technology there is a broad trend of decreasing blank size. As flake size plays such a significant role in cutting edge efficiency, we can therefore begin to explain the trend observed in Fig 2. It is important to note however, that not all aspects of blank size determine variation in efficiency. Rather, specific morphological attributes such as thickness, width and platform size appear to be the key variables.

## Skill

Finally, we seek to examine the role of knapping skill on the cutting edge efficiency of each reduction sequence. As mentioned in the methods section, bipolar reduction was conducted by the expert knapper only due to the extremely low skill required, and punch and pressure blade reduction was conducted by the expert knapper only owing to the high level of skill required. In this section therefore, we examine the influence of skill level on the cutting edge efficiency of multiplatform, discoidal, biface, Levallois and prismatic blade core reduction (Table 3).

**Table 3:** Number of blanks, mass of waste and cutting edge per gram values between the expert knapper (N = 2 for all five technologies) and intermediate knapper (N = 5 for all five technologies) for each technology.

Reduction Method	Skill Level	Mean number of blanks	Mean mass of waste (g)	Mean cutting edge per gram (mm/g)
Multiplatform	Expert	67	62.5	12.43
	Intermediate	118	197.04	18.69
Discoidal	Expert	110	95.6	18.23
	Intermediate	104.6	227.32	17.48
Biface	Expert	223	202.71	21.06
	Intermediate	178.4	140.19	18.73
Levallois	Expert	140.5	145.55	22.68
	Intermediate	131.4	261.46	21.50
Prismatic Blade	Expert	156	144.15	26.09
	Intermediate	125.8	283.48	21.01

In all bar one reduction sequence (biface), the expert knapper produced much less waste debitage compared with the intermediate knapper. However, this represents only a relative assessment of raw material efficiency. Therefore, two-sample t-tests were used to explore any significant variation between the cutting edge per gram output of the expert and intermediate knappers for each of the four reduction strategies. There was no significant difference between the cutting edge per gram efficiency of multiplatform (*t* = -1.86; *df* = 1, 5; *p* = 0.12), discoidal (*t* = 0.25; *df* = 1, 5; *p* = 0.82), biface (*t* = 1.12; *df* = 1, 5; *p* = 0.31) or Levallois (*t* = 0.43; *df* = 1, 5; *p* = 0.69) knapping. Comparatively, the expert knapper produced significantly more cutting edge per gram for the blade core iterations compared with the intermediate knapper (*t* = 4.76; *df* = 1, 5; *p* = 0.005).

This does not mean that the intermediate knapper necessarily executed the multiplatform, discoidal, biface and Levallois reduction strategies as effectively as the expert knapper however. For example, general observations of Levallois flake size and shape as well as the number of recurrent Levallois flakes successfully removed suggest that the expert knapper more effectively performed the Levallois experiments. What can be concluded is that the cutting edge efficiency of multiplatform, discoidal, biface and Levallois technology is less sensitive to reductions in knapper skill compared to prismatic blade technology. In other words, equivalent cutting edge is produced from these knapping strategies regardless of whether a less skilful knapper imperfectly executes the reduction sequence and produces less technologically typical flakes, such as broad dihedral flakes for discoidal knapping, or thin and large Levallois flakes. It is possible that prismatic blade knapping was more efficiently performed by the expert knapper because the desire for thin and long flakes in this technology increases the likelihood of snaps and hinge or step terminations, the correction of which can waste valuable raw material. The elongate core face typical of prismatic blade reduction also makes remedying such mistakes more difficult and costly in terms of raw material usage, as these mistakes tend to be further from the platform. The significant difference in output between intermediate and expert prismatic blade knappers also raises the possibility that prismatic blade technology lends itself to greater craft specialisation compared with the other technologies examined.

When considering the blade reductions performed by the expert knapper only, Fig 3 shows that all three versions of blade reduction possessed approximately equivalent cutting edge efficiency. Therefore, it appears that gains in efficiency are offered by any blade reduction technology as long as they are conducted by expert knappers. This discrepancy between intermediate and expert also serves to further reinforce the interpretation that little change in efficiency occurred at the Middle to Upper Palaeolithic transition.

## Discussion and Conclusions

This study investigated the raw material efficiency of eight different lithic core technologies by measuring the ratio of cutting edge length to original core mass. The results garnered from the 5930 blanks produced in the experiments revealed a gradual upward trend in cutting edge efficiency through the sequence of bipolar, multiplatform, discoidal, biface, Levallois, prismatic blade, punch blade and pressure blade technologies (Fig 2). Interestingly, no statistically significant differences occurred among the individual reduction strategies. Any changes in cutting edge efficiency occurring throughout the evolution of stone tool technology therefore appear to be gradual. These changes were only perceptible when viewing prehistoric tool-kits on a broader-scale, by grouping each technology into their broad time periods. This revealed a significant difference between the raw material efficiency of the technologies typically made in the Lower Palaeolithic and those typically made in the Middle Palaeolithic. In contrast, no significant difference occurred among the Middle Palaeolithic technologies and those in this sample that were made in the Upper Palaeolithic, Epipalaeolithic, Mesolithic and Neolithic.

The fact that the cutting edge lengths per gram of all eight technologies were statistically indistinguishable highlights the shortfalls of comparing only two lithic technologies at a time as was done in all previous comparisons. An experimental comparison of two technologies is likely to confirm the null hypothesis that no significant difference in cutting edge efficiency exists. By examining eight technologies which broadly span the evolution of lithic technology from the Oldowan to the Neolithic, we identified statistically significant trends in cutting edge efficiency over time. The null hypothesis, that no significant differences in cutting edge per gram of core occur among the eight examined technologies, can therefore be rejected as the technologies ascribed to the Middle Palaeolithic were more efficient than those ascribed to the Lower Palaeolithic.

While Eren et al. [61] sought to examine the Middle to Upper Palaeolithic transition using discoidal and prismatic blade core technology, more recent dates of prismatic blades situate their emergence long before the Upper Palaeolithic [54–57]. Meanwhile, discoidal technology is better situated in the Lower Palaeolithic [4, 5, 93]. Therefore, while they found prismatic blades to be no more efficient than discoidal flakes, what was really being compared was the Lower and Middle Palaeolithic. We can therefore, for the first time, conclude that it is unlikely that the Middle to Upper Palaeolithic transition was accompanied by an increase in the raw material efficiency of the available toolkits.

It should of course be noted that there are far more than eight lithic technologies, but with all other comparisons of core efficiency comparing no more than two technologies, we offer a step in the right direction. By selecting representative technologies from different periods, we aimed to capture much of the variation occurring over the sweep of human evolution. Ascribing certain lithic technologies to certain time periods, as we have attempted here, is a difficult task as the picture of evolution in lithic technology becomes increasingly branching and multidirectional. This was done in order to provide a broad-scale picture of changes in efficiency, but should be considered with caution as new sites and dates arise. Similarly, we do not wish to give the impression that the ascending trend observed in Fig 2 in any way suggests cutting edge efficiency evolved in a linear fashion. Rather, much like our biological evolution, it is likely that the evolution of lithic technology and cutting edge production rates was equally complex, branching and recursive. Any apparent linear trend is merely an artefact of taking such a chronologically broad view of lithic technology. We hope this present study offers a broad and exploratory assessment that could be used as a platform for more focussed and site-specific comparisons of raw material efficiency.

We additionally sought to identify attributes of individual flakes which maximise their ratio of cutting edge to flake mass. The measurements and qualitative attributes of 488 complete flakes revealed that the most efficient flakes are those that are small, thin and narrow, with diffuse bulbs, small platforms, feather terminations and extensive platform preparation. Interestingly, elongation and exterior platform angle had minimal to negligible effects on raw material efficiency. It is therefore no surprise that the pressure blade cores performed the most efficiently of all eight technologies under investigation, as pressure blade manufacture involves taking the notion of platform preparation and isolation, key factors in minimising flake thickness, width and bulb thickness, to the extreme. These findings also have significant implications for assemblages with flakes possessing these optimal attributes. Microblade and microlithic technologies, sometimes made via the pressure technique, typically possess these traits and may therefore represent an optimisation of lithic technology geared towards maximising efficiency, whether a conscious attempt or a persistent behavioural adaptation. For example, microliths have been linked to periods of environmental, demographic or social stress, making such technologies likely strategies for offsetting risk in scenarios of raw material scarcity or environmental stress [138]. Further research is required to investigate this possible association between lithic raw material scarcity and strategies which optimise the cutting edge efficiency of flakes.

These results suggest that throughout our biological and cognitive evolution, the major evolution in cutting edge efficiency likely occurred around the transition from the Lower Palaeolithic to the Middle Palaeolithic. The transition from the Middle to Upper Palaeolithic on the other hand, does not appear to be accompanied by a toolkit-wide increase in cutting edge efficiency. This means that the toolkits of the Neanderthals and their contemporaneous *Homo sapiens* exhibited comparable degrees of raw material efficiency. However, we demonstrated that pressure blade technology involved the highest cutting edge efficiency of the eight technologies investigated. Therefore after this transition, during parts of the Upper Palaeolithic, Epipalaeolithic, Mesolithic and Neolithic, *Homo sapiens* continued developing their blade core technology to produce more efficient blank technologies. Minimising flake thickness, bulb thickness and flake width was achieved via specialised blade knapping techniques like pressure knapping, rather than direct percussion. While this technique requires greater investments in preparatory time, through pressure indentor manufacture as well as more intensive platform preparation, it allows for heightened raw material efficiency. Future research is needed to investigate the relationship between heightened investment and raw material efficiency, and model whether these strategies represent an optimisation of the knapping process.

## Supporting Information

S1 Dataset Raw data accompanying S1 Text. (XLSX)

S2 Dataset Flake measurements used within this study. (XLSX)

S1 Text Pilot study testing the efficacy of using a standardised copper billet as an analogue for a range of natural soft hammers. (DOCX)
