# Supplementary material for: Identifying Major Transitions in the Evolution of Lithic Cutting Edge Production Rates
Source: PLoS One. 2016 Dec 9;11(12):e0167244. doi: 10.1371/journal.pone.0167244 (PMC5147885; doi:10.1371/journal.pone.0167244)
Supplement: S1 Text — (DOCX) [file pone.0167244.s003.docx]

**Identifying Major Transitions in the Evolution of Lithic Cutting Edge Production Rates**

Antoine Muller* and Chris Clarkson

* Corresponding author

E-mail: [antoine.muller@uqconnect.edu.au](mailto:antoine.muller@uqconnect.edu.au) (AM)

**S1 Text. Pilot study testing the efficacy of using a standardised copper billet as an analogue for a range of natural soft hammers.**

This document and accompanying dataset (S2 Dataset) outlines the pilot study conducted to support the use of a standardised copper billet during the reduction experiments on which this paper is based. While others have identified copper billets as analogous to natural soft hammers in terms of hardness and performance [1-4] a copper-headed billet would not have been available to Palaeolithic knappers. Archaeological, experimental and ethnographic evidence demonstrates the use of soft stones, antler, bone, wood and other relatively soft materials instead.

Of the eight knapping technologies under examination here, all bar one were typically knapped in prehistory using soft percussors. Bipolar knapping however, involves the use of a hard stone and anvil. Accordingly, a hard stone was used during the bipolar knapping sequence. As mentioned in the main text, a single copper billet was used as an analogue for the broad range of soft percussors routinely used in the archaeological record for the multiplatform, discoidal, biface, Levallois and prismatic blade reductions.

To test the efficacy of using this standardised copper billet, a pilot study was conducted to compare the morphology of flakes produced by soft stone (sandstone), antler and copper percussors of similar masses (307, 319 and 301g respectively). A single cobble of obsidian (2500g) was knapped using the single platform reduction method. Obsidian was used as it is one of the most homogeneously fine-grained knappable materials in the hopes that any morphological differences would be accentuated. The three percussors were used in sequence, with the soft stone percussor being used to remove one flake, followed by the copper, then the antler and so on until the core was exhausted (1.53g) after 37 iterations of this sequence. This was done so that the changing morphology of the core would have negligible impacts on the three samples.

Mass, length, proximal width, medial width, distal width, thickness, platform width, platform thickness and external platform angle (EPA) were all measured. A geometric mean of key size related attributes (mass, length, medial width and thickness) was used as a comprehensive and scale independent measure of flake size. Convergence was calculated by subtracting distal width from proximal width, and elongation represents length as a ratio of medial width. Platform type was additionally recorded. The results of these analyses can be seen below (Table 1) and the underlying dataset is available as S2 Dataset.

**Table 1.** Morphological attributes of the 111 flakes measured as part of this pilot study.

| Median Values | Soft Stone (N=37) | Copper (N=37) | Antler (N=37) | Kruskal-Wallis Test |
| --- | --- | --- | --- | --- |
| Mass (g) | 8.03 | 11.53 | 23.36 | H=7.37; p=**0.025** |
| Size Geometric Mean | 15.89 | 18.06 | 24.96 | H=6.32; p=**0.042** |
| Platform Width (mm) | 19.99 | 19.71 | 23.81 | H=3.50; p=0.17 |
| Platform Thickness (mm) | 7.03 | 7.55 | 8.99 | H=5.39; p=0.068 |
| EPA (^o^) | 72 | 72 | 72 | H=2.30; p=0.32 |
| Convergence (mm) | 2.88 | 0.57 | 0.54 | H=1.09; p=0.58 |
| Elongation | 2.03 | 1.91 | 1.72 | H=2.16; p=0.34 |
| Length:Thickness | 7.12 | 6.59 | 5.91 | H=8.51; p=**0.014** |
| Width:Thickness | 3.93 | 3.50 | 3.32 | H=5.19; p=0.075 |

For the three morphological attributes that displayed significant differences (mass, the geometric mean of size attributes and the length to thickness ratio), a series of Mann-Whitney U tests with Bonferroni corrections reveals that the significant differences are between the soft stone and antler samples (p = 0.028, 0.048 and 0.013 respectively). Meanwhile, no significant differences exist between either the copper and soft stone or copper and antler samples for these three morphological attributes. Importantly, in eight out of nine of these morphological attributes, the copper billet produced flakes with median values between the soft stone and antler samples. In terms of platform type, Table 2 again shows that the copper billet performed on par with, or between the soft stone and antler hammers.

**Table 2.** Proportion of different platform types produced by the different hammers.

|  | Soft Stone (N=37) | Copper (N=37) | Antler (N=37) |
| --- | --- | --- | --- |
| Plain | 29 | 29 | 27 |
| Dihedral | 3 | 4 | 7 |
| Focalised | 5 | 4 | 3 |

We can tentatively conclude therefore, that not only is the morphology of flakes produced by the copper percussor statistically indistinguishable from the morphology of flakes produced by soft stone and antler percussors, but it also acts to consistently average the variability between these two natural soft hammers. It is important to note that only in very rare instances (e.g. [5]) is it possible to determine with certainty which particular soft hammer has been used in the archaeological record. Therefore, using a specific soft hammer in experiments because it may have been used by prehistoric knappers is an inadequate methodological justification and may result in flakes with statistically dissimilar morphologies between the experimental and archaeological assemblages.

In the majority of cases, where the particular soft hammer used is unknown, we therefore recommend the use of a copper billet that not only offers standardisation and experimental control, but also a more reliable analogue for the wide range of soft hammers available. It is for these reasons that we use a copper billet for the majority of reduction sequences performed for this paper.

**References**

1. Clark JE. Stoneworkers’ approaches to replicating prismatic blades In: Desrosiers PM, editor. The Emergence of Pressure Blade Making: From Origin to Modern Experimentation. New York: Springer; 2012. p. 43-135.

2. Crabtree DE. Notes on experiments in flintknapping. Tebiwa. 1967;10:60-73.

3. Crabtree DE. Mesoamerican polyhedral cores and prismatic blades. Am Antiq. 1968;33(4):446-78.

4. Sheets PD, Muto GR. Pressure blades and total cutting edge: an experiment in lithic technology. Science. 1972;175(4022).

5. Langejans GHJ. Middle Stone Age pièces esquillées from Sibudu Cave, South Africa: an initial micro-residue study. Journal of Archaeological Science. 2012;39:1694-704.
